# Prognostic significance of lymph node ratio in esophageal squamous cell carcinoma: insights from the South Asian population

**DOI:** 10.3389/fonc.2024.1430876

**Published:** 2025-01-17

**Authors:** Sajida Qureshi, Waqas Ahmad Abbasi, Hira Abdul Jalil, Saba Mughal, Muhammad Saeed Quraishy

**Affiliations:** ^1^ Dow Medical College, Dow University of Health Sciences, Karachi, Pakistan; ^2^ School of Public Health, Dow University of Health Sciences, Karachi, Pakistan

**Keywords:** esophageal carcinoma, lymph node ratio, prognosis, surgical resection, minimally invasive esophagectomy

## Abstract

**Background:**

Esophageal cancer (EC) is a significant health concern in South Asia, yet data on prognostic factors, such as lymph node ratio (LNR), in this region is limited. This study aims to assess the prognostic significance of LNR in esophageal squamous cell carcinoma (ESCC) patients undergoing concurrent neoadjuvant therapy followed by minimally invasive esophagectomy (MIE).

**Methods:**

This retrospective study analyzed the clinical data of ESCC patients who underwent concurrent neoadjuvant therapy followed by MIE at Dr. Ruth K. M. Pfau Civil Hospital from 2019 to 2023. Lymph node ratios were derived and patients were categorized into three groups: LNR 0, LNR low (≤ 0.1), and LNR high (>0.1). Patient characteristics were compared along with lymph node groups, and survival outcomes were analyzed using the Kruskal Wallis and Chi-square/Fisher exact test, Pearson correlation, Kaplan-Meier (KM) estimates, and Cox regression models.

**Results:**

Among the 47 patients, 15 (31.9%) deaths were observed. Patients with a high LNR had a higher mortality rate (70%) compared to those with a low LNR (41.7%) and 0 LNR (12%) (p = 0.002). Additionally, patients with a high LNR (>0.1) were associated with poorer overall survival (OS) (30.0% vs. 58.3% vs. 88.0%, p < 0.001). A significant correlation was also observed between LNR and the number of metastatic lymph nodes (correlation coefficient = 0.928, p < 0.001).

**Conclusion:**

Our findings demonstrate that high LNR emerged as an independent prognostic factor in ESCC patients undergoing concurrent neoadjuvant therapy followed by MIE.

## 1 Introduction

Esophageal cancer (EC) has emerged as a significant public health challenge in Pakistan, contributing considerably to the overall disease burden as the 4th most prevalent cancer, with an occurrence rate of 5168 per 100,000 population across all ages and genders (1, 2). Esophageal squamous cell carcinoma (ESCC) comprises 90% of cases globally, with a predominant occurrence in the Asian region. In Pakistan, the majority of EC cases belong to the ESCC subtype (3). Despite notable advancements in treatment modalities and staging methodologies, the malignancy’s overall survival (OS) remains low (4). Within the spectrum of prognostic factors, the significance of lymph node (LN) metastasis, particularly the number of metastatic LN, has been highlighted in the staging systems of entities like the American Joint Committee on Cancer (AJCC) and the Union for International Cancer Control (UICC) since 2009 (5–7). Furthermore, the quantity of LNs removed during surgery has appeared as a critical determinant, affecting patient prognosis, as evidenced by numerous studies (8, 9). In response to this nuanced landscape, the concept of the lymph node ratio (LNR), denoting the ratio of positive LNs to the total number of removed LNs during surgical resection, has gathered noteworthy attention as an essential prognostic factor in gastrointestinal cancers, including EC (10–12). Considering the limited exploration of LNR in EC patients within South Asian population, specifically Pakistan, our goal is to provide valuable data through this retrospective cohort analysis.

Therefore, we aim to thoroughly investigate the prognostic implications of LNR in ESCC patients who underwent minimally invasive esophagectomy (MIE), with directly assessing its impact on the survival.

## 2 Materials and methods

### 2.1 Study design and site

IRB approval was obtained (IRB-3388/DUHS) and we retrospectively retrieved and reviewed the medical records of EC patients at the Department of Upper GI Surgery, Surgery Unit-I, Dr. Ruth KM Pfau Civil Hospital in Karachi, which is one of the biggest government sector tertiary care settings.

### 2.2 Sample size, inclusion, and exclusion criteria

Biopsy-proven ESCC patients who underwent concurrent neoadjuvant therapy followed by MIE from 2019 to 2023, completing a minimum 6-month follow-up period, were included. Exclusion criteria involved patients with abandoned surgery due to complications, cases that were converted to open surgery, incomplete records, and those who were lost to follow-up.

### 2.3 Surgical procedure, data collection and analysis

Filled proformas were utilized to retrieve comprehensive patient data, encompassing all details, from the time of admission to their last follow-up. This approach ensured strict adherence to our inclusion and exclusion criteria, with any incomplete records being excluded to maintain data integrity. Data collection included information on the concurrent neoadjuvant therapy regimen, the type of lymphadenectomy performed, operative parameters such as operative time (in minutes) and estimated blood loss (in mL) to assess the quality of resection, as well as short-term postoperative complications and 30-day mortality to evaluate the immediate impact of surgical interventions. None of the patients in this cohort received postoperative adjuvant therapy. This is consistent with standard clinical practice, as adjuvant therapy is not routinely recommended for ESCC patients who undergo neoadjuvant treatment followed by R0 resection (13, 14).

Additionally, it’s noteworthy that all included patients had undergone pathological and biopsy assessments at the same laboratory facility, ensuring standardized evaluation. Our data retrieval method was complemented by a systematic approach to data quality control approach, including thorough reviews of all medical records, pathology reports, and surgical notes. Weekly follow-up clinic data was reviewed to assess survival outcomes comprehensively. Patients who missed their weekly follow-ups were contacted via tele-service, and the final survival data was compiled based on their last recorded follow-up. In this cohort, we then derived LNR using the data of the resected number of LNs and positive number of LNs, classifying patients into three groups: LNR 0, LNR low (≤ 0.1), and LNR high (>0.1).

Data analysis was done using SPSS version 27. Pearson correlation analysis was performed to determine the correlation between LNR and the number of positive LNs. Descriptive statistics such as frequency, percentage for categorical variables and median, range, interquartile range for quantitative variables were reported. Associations of clinicopathological characteristics of patients were examined with LNR (LNR 0, LNR low, and LNR high) using Kruskal Wallis and Chi-square/Fisher exact test. The OS was measured from the date of diagnosis to the last date of follow-up. Survival curves were plotted by using the Kaplan-Meier estimates and differences were compared with log-rank test. A Cox proportional hazards model was used for univariate and multivariate regression analysis. Covariates with p <0.25 in univariate analysis were considered for multivariate analysis. Hazard ratio (HR) and 95% confidence interval (95% CI) were reported. Statistical significance was considered at two-sided p-value < 0.05.

## 3 Results

### 3.1 Patient characteristics and outcomes

This analysis included a total of 47 biopsy-proven ESCC patients who underwent concurrent neoadjuvant therapy followed by MIE between 2019 and 2023. The median age of the ESCC patients in our study cohort was 43 years, ranging from 22 to 72 years, with 20 (42.6%) males and 27 (57.4%) females. Among them, 31 (66%) patients had moderately differentiated tumor cells, 8 (17.0%) showed well-differentiation, and 6 (12.8%) were poorly differentiated. Additionally, 27 (57.4%) patients had tumor lengths between 5 to 10 cm, while 18 (38.3%) had tumor lengths <5 cm, and 2 (4.3%) had tumor lengths >10 cm. Notably, 23 (48.9%) patients were diagnosed with stage III cancer, while 15 (31.9%) were diagnosed with stage IV cancer, indicating advanced progression within the cohort. According to LNR groups, 10 (21.3%) had high LNR (>0.1), 12 (25.5%) had low LNR and 25 (53.2%) belonged to 0 LNR group.

All patients underwent a standardized concurrent neoadjuvant therapy regimen prior to the definitive procedure. This regimen consisted of 4 cycles of chemotherapy with carboplatin, paclitaxel, cisplatin, and fluorouracil, combined with 25-28 sessions of radiotherapy (45 Gy). The median operative time of the cohort was 320 minutes (range 180–485 minutes), with a median estimated blood loss of 100 mL (range 50–200 mL). Postoperative complications occurred in 19.1% of patients, including chest infection (6.4%, n=3), voice changes (4.3%, n=2), tachycardia (2.1%, n=1), wound infection (2.1%, n=1), pneumothorax (2.1%, n=1), and pleural effusion (2.1%, n=1). The 30-day mortality rate was 4.3% (n=2) in the cohort of 47 patients.

15 (31.9%) deaths were observed, and it was noted that patients who have high LNR (70%) faced an event (death) more as compared to those who have low LNR (41.7%) and 0 LNR (12%) (p=0.002). Furthermore, T stage was also significantly associated with the LNR groups (p=0.026). A detailed comparison between LNR groups and clinicopathological characteristics is summarized in **Table 1**.

**Table 1:** Demographic and clinicopathological characteristics of esophageal cancer patients undergoing MIE according to LNR groups (n=47).

Characteristics	Total (n=47)	LNR 0 (n=25)	LNR Low (n=12)	LNR High (n=10)	p-value*
	Median (Q1-Q3)	Median (Q1-Q3)	Median (Q1-Q3)	Median (Q1-Q3)	
Age in years	43 (33 - 55)	43 (34 - 52)	41 (32 - 60)	45 (33 - 57)	0.997
Time in months	12 (8 -19)	12 (10 - 17)	18 (8 - 21)	8 (11 - 13)	0.324
Neutrophil to lymphocyte ratio (NLR)	3.0 (2.2 - 4.0)	3.0 (2.2 - 3.7)	2.7 (2.1 - 4.1)	4.0 (2.4 - 6.4)	0.469
Platelet to lymphocyte ratio (PLR)	176 (140 - 214)	188 (142 - 218)	176 (116 - 200)	152 (140 - 249)	0.626
Lymphocyte monocyte ratio (LMR)	3.8 (2.3 - 4.8)	3.9 (1.9 - 6.4)	3.8 (2.5 - 4.5)	3.3 (2.3 - 4.2)	0.806
Platelet to RDW ratio (PRR)	5.5 (3.8 - 8.3)	6.3 (4.2 - 8.8)	4.3 (2.9 - 7.6)	5.6 (3.4- 11.2)	0.260
	n(%)	n(%)	n(%)	n(%)	
Gender					
Male	20 (42.6)	14 (56.0)	4 (33.3)	2 (20.0)	0.114
Female	27 (57.4)	11 (44.0)	8 (66.7)	8 (80.0)	
Grade of differentiation					
Well differentiated	8 (17.0)	5 (20.0)	2 (16.7)	1 (10.0)	NA
Moderately differentiated	31 (66.0)	13 (52.0)	9 (75.0)	9 (90.0)	
Poorly differentiated	6 (12.8)	5 (20.0)	1 (8.3)	0 (0)	
None	2 (4.3)	2 (8.0)	0 (0.0)	0 (0)	
Tumor length					
< 5 cm	18 (38.3)	11 (44.0)	5 (41.7)	2 (20.0)	NA
5 - 10 cm	27 (57.4)	13 (52.0)	6 (50.0)	8 (80.0)	
> 10 cm	2 (4.3)	1 (4.0)	1 (8.3)	0 (0)	
T stage					
To	13 (27.7)	11 (44.0)	1 (8.3)	1 (10.0)	0.026
T1	18 (38.3)	10 (40.0)	5 (41.7)	3 (30.0)	
T2	11 (23.4)	4 (16.0)	4 (33.3)	3 (30.0)	
T3	5 (10.6)	0 (0)	2 (16.7)	3 (30.0)	
N stage					
N0	24 (51.1)	24 (96.0)	0 (0)	0 (0)	NA
N1	16 (34.0)	0 (0)	12 (100.0)	4 (40.0)	
N2	6 (12.8)	1 (4.0)	0 (0)	5 (50.0)	
N3	1 (2.1)	0 (0)	0 (0)	1 (10.0)	
Clinical stage					
I - II	9 (19.1)	7 (28.0)	2 (16.7)	0 (0)	0.11
III	23 (48.9)	8 (32.0)	7 (58.3)	8 (80.0)	
IV	15 (31.9)	10 (40.0)	3 (25.0)	2 (20.0)	
Patient Status					
Death	15 (31.9)	3 (12.0)	5 (41.7)	7 (70.0)	0.002
Alive	32 (68.1)	22 (8.0)	7 (58.3)	3 (30.0)	

*p-value was calculated by Kruskal Wallis test and Chi-square/Fisher exact test.

NA represents not applicable.

### 3.2 Correlation between LNR and the number of positive LNs

**Figure 1:**
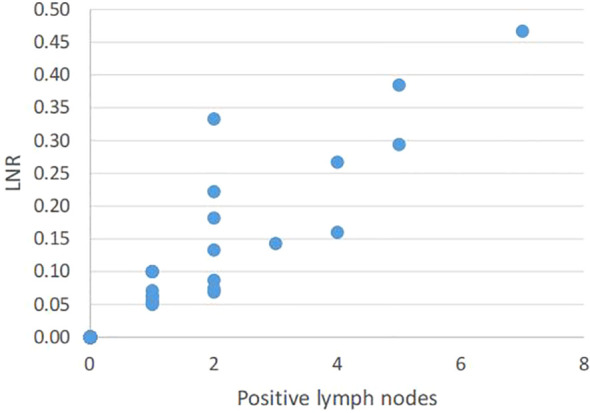
Relationship between positive lymph nodes and LNR.

In 47 patients, a total of 715 LNs were removed during surgery. For lower and mid esophageal tumors, a two-field lymphadenectomy was performed, removing lymph nodes from the mediastinal and abdominal regions, while for upper esophageal tumors, a three-field lymphadenectomy was conducted to include nodes from the cervical, mediastinal, and abdominal regions, aiming for comprehensive nodal clearance. Among the harvested LNs, 51 (7.13%) were identified as metastatic nodes. The median number of LNs harvested per person during surgery was 15 (range, 4 to 35). Pearson correlation analysis showed that there was a significant correlation between LNR and the number of metastatic LNs (correlation coefficient = 0.928, p<0.001) (**Figure 1**).

### 3.3 Survival analysis and prognostic factors

**Figure 2:**
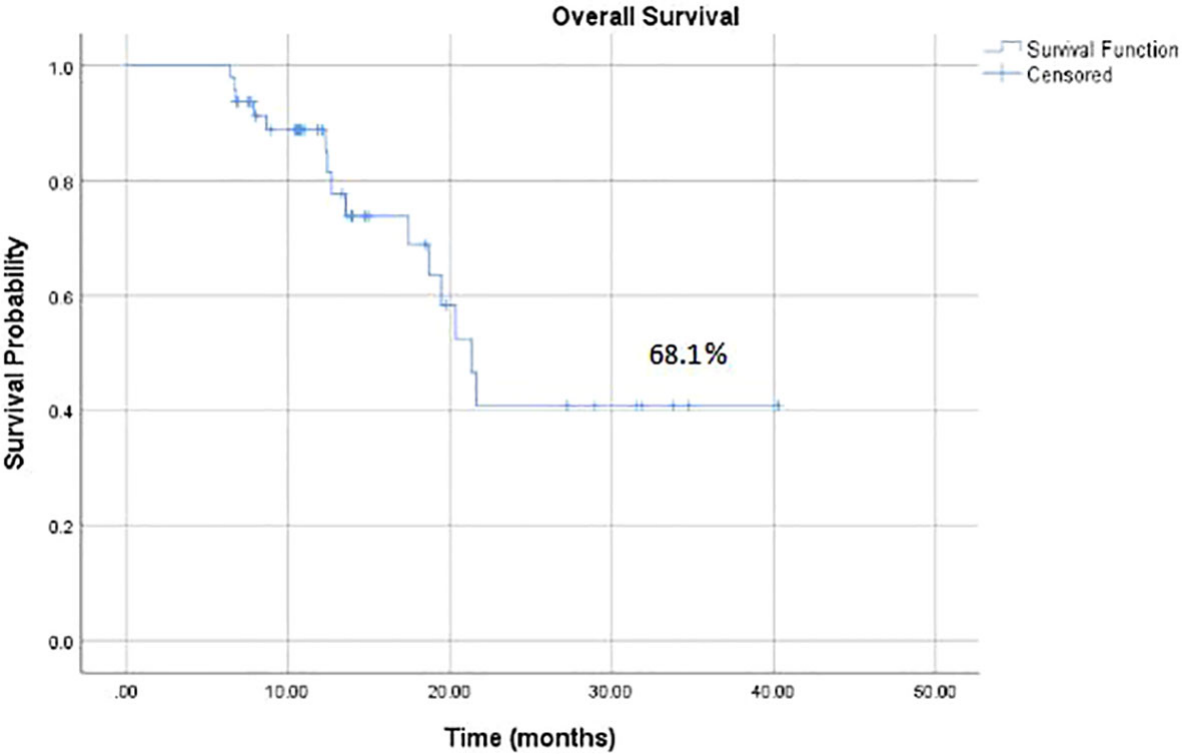
Kaplan–Meier estimate of OS.

**Figure 3:**
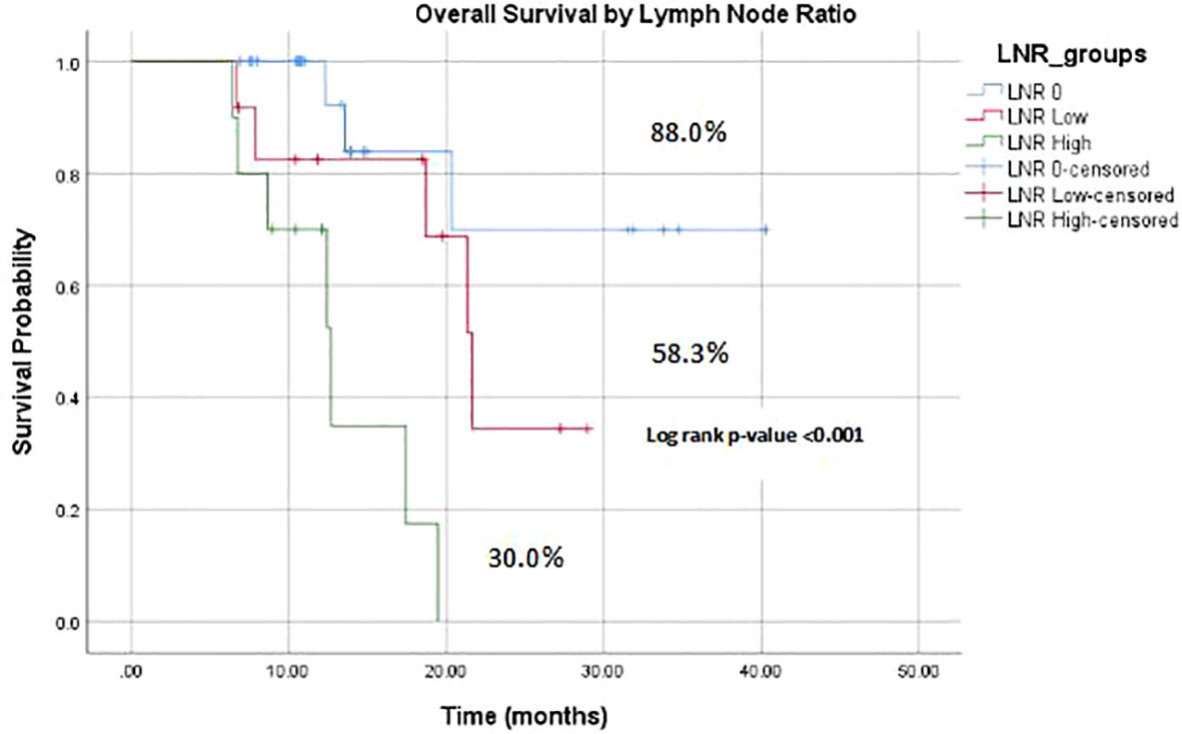
Kaplan–Meier estimate of OS based on LNR groups.

Median follow-up time was 12 months, which ranged between 7 – 40 months. The median OS was 11 months and OS rate of patients was 32 (68.1%) (**Figure 2**). KM estimates of OS are plotted in **Figure 2**. It was further noted that high LNR (OS: 30% vs. 58.3% vs. 88.0%, log-rank p-value=0.001) were significantly associated with poor OS of patients (**Figure 3**). Univariate Cox regression model revealed that patients with high LNR >0.1 (HR=12.59, 95% CI: 2.90-54.46, p-value=0.004) were significantly associated with decreased survival as compared to those who had LNR 0. Multivariate model was adjusted for those covariates who had p-value<0.25 in univariate analysis. It was observed that high LNR > 0.1 (HR = 11.51, 95% CI: 2.59–51.06, p-value=0.001) was significantly affecting the OS of patients (**Table 2**).

**Table 2:** Univariate and multivariate cox proportional hazards model for the risk factors associated with mortality among patients with esophageal cancer.

Characteristics	Survival	Univariate		Multivariate	
	(%)	HR (95% CI)	p value	HR (95% CI)	p value
Age in years		0.98 (0.94-1.02)	0.310	–	
Neutrophil to lymphocyte ratio (NLR)		0.99 (0.83-1.18)	0.954	–	
Platelet to lymphocyte ratio (PLR)		0.99 (0.99-1.00)	0.881	–	
Lymphocyte monocyte ratio (LMR)		1.07 (0.83-1.37)	0.601	–	
Platelet to RDW ratio (PRR)		1.06 (0.95-1.18)	0.290	–	
Gender					
Male	80.0	Ref		Ref	
Female	59.3	2.34 (0.73-7.44)	0.149	1.80 (0.55-5.84)	0.328
Grade of differentiation					
Well differentiated	62.5	Ref			
Moderately differentiated	67.7	1.15 (0.31-4.22)	0.827	–	
Poorly differentiated	66.7	1.09 (0.18-6.62)	0.922		
Tumor length					
< 5 cm	77.8	Ref			
5 - 10 cm	63.0	1.14 (0.35-3.71)	0.820	–	
> 10 cm	50.0	2.27 (0.24-20.92)	0.467		
T stage					
To	84.6	Ref			
T1	66.7	1.12 (0.21-5.76	0.892	–	
T2	63.6	1.68 (0.30-9.29)	0.549		
T3	40.0	2.26 (0.36-13.95)	0.378		
Clinical stage					
III	56.5	Ref			
IV	66.7	0.58 (0.19-1.73)	0.335	–	
Lymph node ratio					
0	88.0	Ref		Ref	
Low (≤0.1)	58.3	2.53 (0.60-10.67)	0.204	2.51 (0.59-10.57)	0.209
High (>0.1)	30.0	12.59 (2.90-54.46)	0.001	11.51 (2.59-51.06)	0.001

Univariate cox proportional hazards model was applied for all independent prognostic variables and Multivariate cox proportional hazard model was adjusted for whose p-value<0.25 in univariate model (gender and lymph node rati0), HR=hazard ratio, CI=confidence interval.

## 4 Discussion

Surgical resection remains the primary treatment for carcinoma esophagus, but despite advances in techniques and lymphadenectomy, overall survival rates remain unsatisfactory (15). Lymph nodal involvement is a crucial prognostic factor in EC, consistently associated with a poorer prognosis (16–18). Previous studies on various cancers, including gastric, breast, and pancreatic, have also confirmed the association between high LNR and low survival rates (19–21). However, limited data for EC concerning LNR in the South Asian population, specifically Pakistani population, makes direct comparisons with previous literature quite challenging.

LNR is considered more useful than just the number of metastatic LNs (19). A meta-analysis by Song et al. reviewed 14 studies from Western Asia, revealing a significant association between high LNR and poor OS (22). Interestingly, no significant difference within the same study was found in patients from any other population. The prognostic value of LNR in ESCC reflects tumor aggressiveness, while in general, larger negative lymph nodes (LNneg) may signal a stronger immune response. However, the tumor microenvironment (TME), with its greater immunosuppressive role, may have a more significant impact on survival outcomes than LNR or immune function alone. That said, no clear evidence currently link these factors (high LNR, OS, and Immune function) specifically in ESCC (23–25).

Similarly, Jang et al. proposed LNR as a significant prognostic factor in patients with ESCC who underwent neo-adjuvant chemo-radiotherapy followed by surgery, suggesting additional treatment and closer follow-up for patients with a high LNR (26). Another study indicated a relationship between an increased LNR and the worsening of patients’ OS (27). Our results showed a similar trend, and among all deaths observed, it was noticeable via multivariate model analysis that patients who expired were more likely to have a high (>0.1) LNR (p-value=0.001), which establishes a strong base for some future relevance in South Asian patients with similar characteristics (**Table 2**).

Although molecular biomarkers offer higher specificity, LNR has shown superior prognostic value in ESCC, with studies indicating that an LNR-based staging system outperforms the TNM system (28) and predicts survival more accurately, especially in patients with fewer than 15 lymph nodes examined (29). In cancers like colorectal, LNR has been superior to TNM pN categories in predicting outcomes, suggesting it could reduce stage migration and improve prognostic accuracy in ESCC (30–32). Furthermore, LNR has proven to be predictive across various subgroups, including our South Asian cohort, reinforcing its role in survival prediction.

Integrating LNR into established systems such as TNM staging could offer a more refined risk stratification. Our study highlights LNR high (>0.1) as a significant marker of poor survival outcomes, suggesting it could serve as a threshold to guide more aggressive monitoring, closer follow-up, or therapeutic interventions. Conversely, LNR low (≤0.1) indicates a more favorable prognosis, potentially allowing for less intensive surveillance. These thresholds could serve as practical tools to tailor patient management strategies, ensuring high-risk patients receive timely interventions, such as adjuvant therapies. However, validation through larger, multicenter studies is essential to confirm the broader clinical applicability of these thresholds.

Moreover, in terms of OS, our study noted a significant discrepancy among the LNR groups, similar to previous findings where the 2-year survival rates were distinctly different: 79.0% for LNR 0, 54.0% for LNR low, and 9.1% for LNR high groups (26). Our investigation, with a median follow-up time of 12 months (range: 7-40 months), revealed a similar trend across the high, low, and LNR 0 groups (OS: 88.0% vs. 58.3% vs. 30.0%, log-rank p-value= <0.001). Furthermore, while surgical resection quality is known to impact outcomes in esophageal cancer, our analysis indicated that operative parameters such as operative time, blood loss, and postoperative complications aligns with typical outcomes for this type of surgery and patient cohort, reinforcing the role of high LNR as an independent prognostic factor. These results emphasize the prognostic relevance of LNR in discerning survival outcomes among patients undergoing treatment for ESCC.

Additionally, the yield of LNs deciding the ratio, does have a prognostic impact too, and is influenced by several factors, including variations in the extent of lymphadenectomy performed by different surgeons, discrepancies in the submission of specimens, and differences in the methodology of LN retrieval by pathologists, where we did took careful considerations to rule out all biases (33–35). We specifically performed two-field lymphadenectomy in lower and mid ECs, while a three-field lymphadenectomy for upper ECs. Guidelines further suggests that for optimal staging, a minimum of 15 to 23 lymph nodes should be resected (36). In our cohort, the median number of lymph nodes harvested per person during surgery was 15.

Despite the valuable insights gained, a notable limitation of our study is the small sample size (n=47), which may affect the generalizability and statistical power of the results. This constraint reflects the high prevalence of advanced, often unresectable cases in our region at presentation, limiting patient eligibility for surgical procedures and, consequently, reducing available data. As a result, the findings may not be fully representative of the broader ESCC population, and caution is needed when extrapolating the results to other populations or subgroups. These factors could affect the robustness of the conclusions. While larger, multicenter studies would strengthen the analysis, it is important to note that this is the first report from our region exploring LNR as a prognostic factor in ESCC. Therefore, this finding remains significant and lays the foundation for future studies seeking a deeper evaluation of similar prognostic indicators in this population. Further prospective studies with larger sample sizes and comprehensive datasets are required to confirm these results and evaluate the broader applicability of LNR in clinical practice.

In conclusion, our findings underscore high LNR as an independent predictor of OS, with higher values linked to poorer survival. This highlights the value of LNR in prognostic assessments for ESCC patients in South Asia.

## Data Availability

The data analyzed in this study is subject to the following licenses/restrictions: Due to patient confidentiality, the dataset is not publicly accessible. Requests to access these datasets should be directed to SQ, sajida.qureshi@duhs.edu.pk.
